# Supplementary material for: Thymidine rescues ATR kinase inhibitor-induced deoxyuridine contamination in genomic DNA, cell death, and interferon-α/β expression
Source: Cell Rep. Author manuscript; Available in PMC 2022 Nov 9. (PMC9646445; doi:10.1016/j.celrep.2022.111371)
Supplement: 1 [file NIHMS1843664-supplement-1.pdf]

Cell Reports, Volume *40*

## Supplemental information

# Thymidine rescues ATR kinase inhibitor-induced deoxyuridine contamination in genomic DNA, cell death, and interferon-α/β expression

**Norie Sugitani, Frank P. Vendetti, Andrew J. Cipriano, Pinakin Pandya, Joshua J. Deppas, Tatiana N. Moiseeva, Sandra Schamus-Haynes, Yiyang Wang, Drake Palmer, Hatice U. Osmanbeyoglu, Anna Bostwick, Nathaniel W. Snyder, Yi-Nan Gong, Katherine M. Aird, Greg M. Delgoffe, Jan H. Beumer, and Christopher J. Bakkenist**

# Thymidine rescues ATR kinase inhibitor-induced deoxyuridine contamination in genomic DNA, cell death, and interferon-α/β expression

**Short Title: ATR kinase inhibition induces deoxyuridine contamination and IFN-α/β expression**

Norie Sugitani[1], Frank P. Vendetti[1], Andrew J. Cipriano[1], Pinakin Pandya[1], Joshua J. Deppas[2], Tatiana N. Moiseeva[3], Sandra Schamus-Haynes[1], Yiyang Wang[4], Drake Palmer[5], Hatice U. Osmanbeyoglu[5, 6], Anna Bostwick[7], Nathaniel W. Snyder[7], Yi-Nan Gong[4,5], Katherine M. Aird[5, 8], Greg M. Delgoffe[4, 5], Jan H. Beumer[2, 5, 9], and Christopher J. Bakkenist[1, 8, 10*]

## SUPPLEMENTAL FIGURES

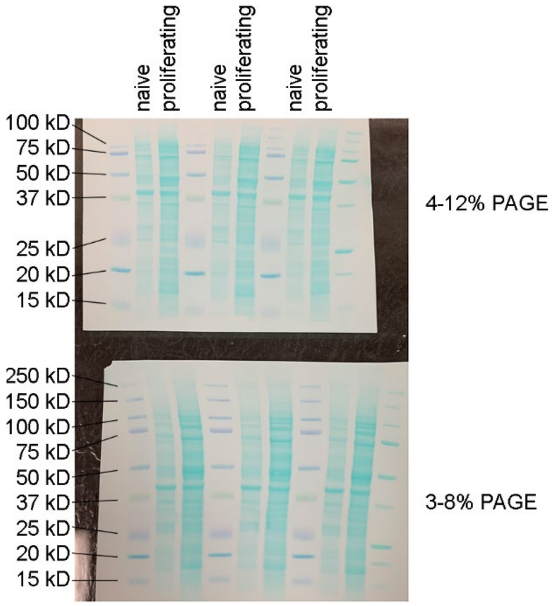


**Figure S1. Blots of CD8⁺ T whole cell extracts, related to Figure 1B.**
Blots of CD8⁺ T whole cell extracts prepared at 0 and 30 h post-activation stained with fast green prior to immunoblotting as shown in Figure 1B. The protein extracts were generated from the same number of naïve and proliferating CD8⁺ T cells.

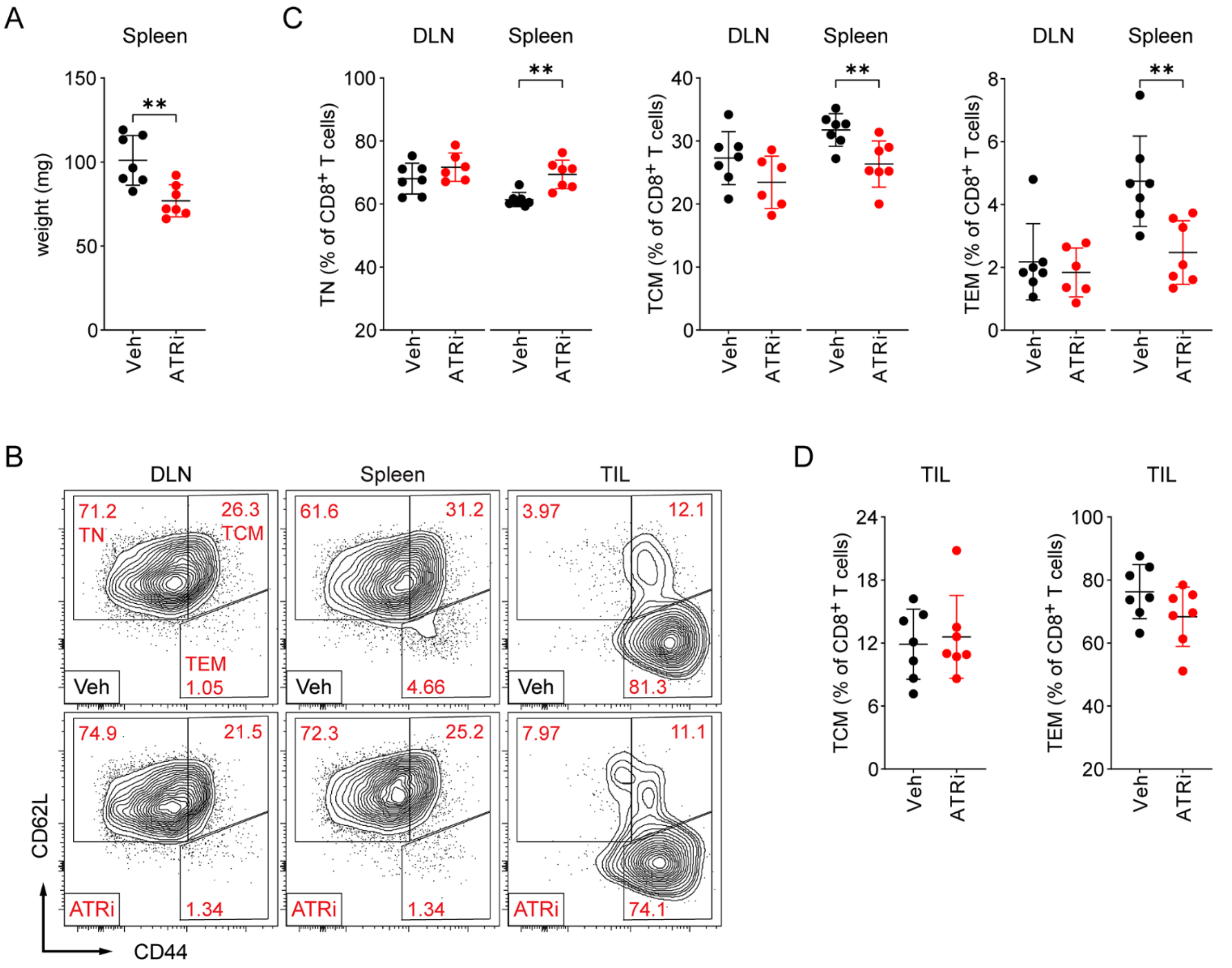


**Figure S2. Quantitation of immune cells in mice, related to Figure 2.**

(A) Weight of whole spleens harvested on day 4 from CT26 tumor bearing mice treated with ATRi (75 mg/kg AZD6738) or vehicle (Veh) on days 1-3.

(B-D) Immunoprofiling of tissues harvested on day 4 from CT26 tumor bearing mice treated with ATRi (75 mg/kg AZD6738) or vehicle (Veh) on days 1-3.

(B) Representative contour plots of $CD8^+$ T cells with naïve (TN, $CD62L^{hi}CD44^{lo}$), central memory (TCM, $CD62L^{hi}CD44^{hi}$), and effector/effector memory (TEM, $CD62L^{lo}CD44^{hi}$) phenotypes in the tumor-draining lymph node (DLN), spleen, and tumor infiltrate (TIL).

(C) Quantitation of TN, TCM, and TEM $CD8^+$ T cells in the DLN and spleen.

(D) Quantitation of TCM and TEM $CD8^+$ T cells in the TIL.

(A-D) n = 7 mice total per group (6 DLN for ATRi) from 2 independent experiments, each with 3-4 mice per group.

(A, C-D) Mean and SD bars shown. **:P<0.01 by two-tailed, unpaired t-test. Brackets not shown for comparisons that were not statistically significant.

A

| Cell Type | Doubling Time (h) | G1 (h) | S (h) | G2 (h) |
|---|---|---|---|---|
| CD8⁺ T cells | 6 | 1.0 ± 0.3 | 4.7 ± 0.3 | 0.3 ± 0.1 |
| B16 | 17.1 | 7.1 ± 1.3 | 9.3 ± 1.3 | 0.7 ± 0.3 |
| 1° Fibroblasts | 29 | 13 ± 3.3 | 8.2 ± 1.8 | 7.5 ± 2.3 |

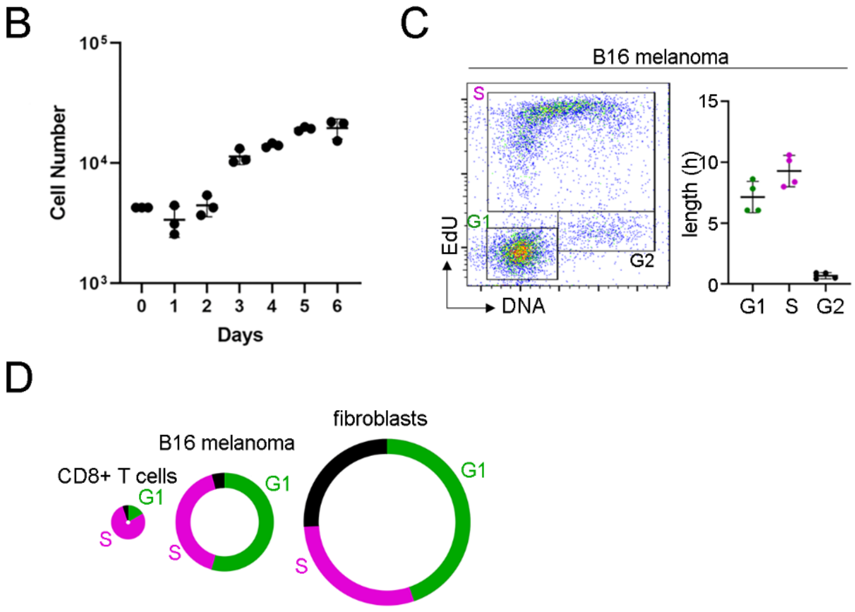


**Figure S3. Proliferation of CD8⁺ T cells, B16 melanoma cells, and fibroblasts, related to Figure 3A,B.**
(A) Summary of doubling times and estimated lengths of G1, S, and G2 phases in CD8⁺ T cells, B16, and primary fibroblasts. The percentage of cells in each cell cycle phase and the doubling time was used to estimate the length of G1, S, and G2/M.
(B) Doubling time of B16 was previously reported (Danciu et al., 2013; Fidler, 1975) while that of primary fibroblasts was calculated with the equation (t2 – t1)/(3.32*log(n2/n1)) using linear part of the growth curve. n1 is the cell number at the time point t1 and n1 is the cell number at the time point t2.
(C) EdU versus DNA histograms of B16. Mean and SD bars shown.
(D) The circumference of the circle represents the doubling time and the lengths of G1, S, and G2/M are drawn to scale.

Danciu, C., Falamas, A., Dehelean, C., Soica, C., Radeke, H., Barbu-Tudoran, L., Bojin, F., Pinzaru, S.C., and Munteanu, M.F. (2013). A characterization of four B16 murine melanoma cell sublines molecular fingerprint and proliferation behavior. Cancer Cell Int *13*, 75. 10.1186/1475-2867-13-75.
Fidler, I.J. (1975). Biological behavior of malignant melanoma cells correlated to their survival in vivo. Cancer research *35*, 218-224.

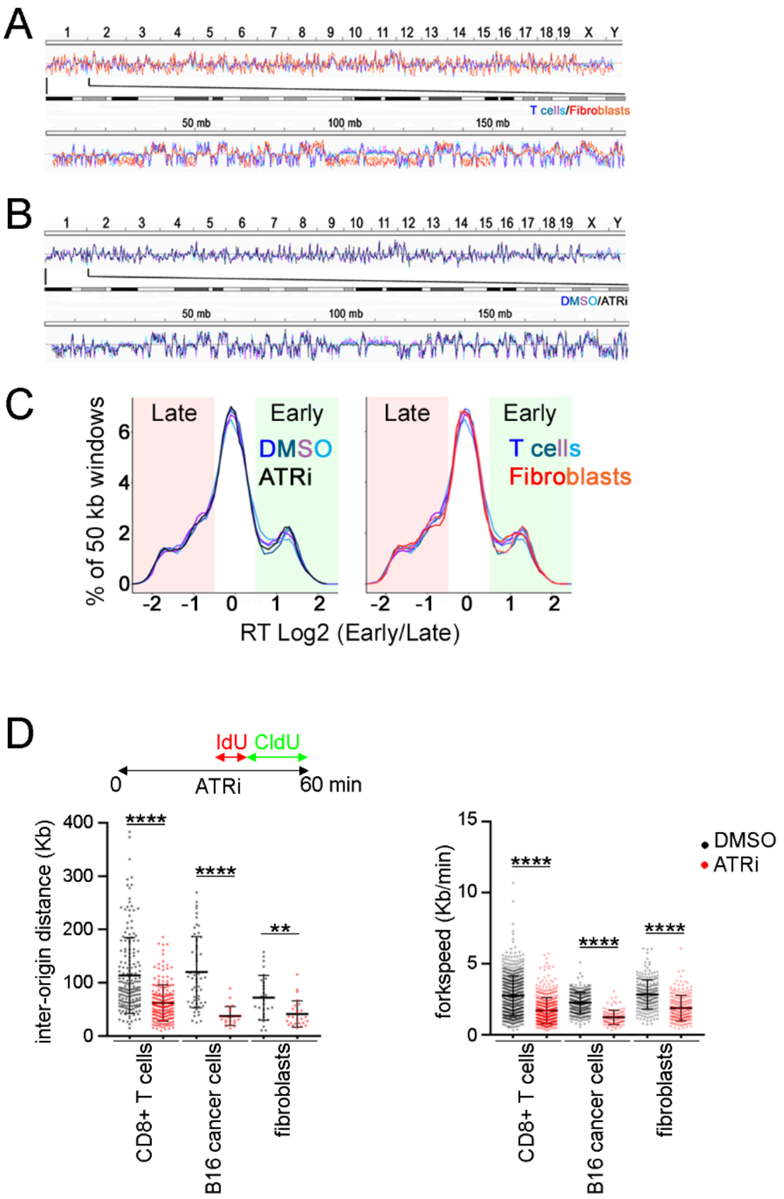


**Figure S4. Analyses of origin firing in CD8$^{+}$ T cells, B16 melanoma cells, and fibroblasts, related to Figure 3D-F.**

(A) Repli-seq analyses of proliferating CD8$^{+}$ T cells (blues) and primary fibroblasts (reds). Upper – whole genome. Lower - chromosome 1. Sequencing data are deposited at GEO (code: GSE183412).

(B) Repli-seq analyses of proliferating CD8$^{+}$ T cells treated with DMSO (blues) or 5 µM AZD6738 (black) for 1 h.

(C) Genome-wide distribution of the replication timing of 50 kb genomic windows determined by repli-seq. Comparison of proliferating CD8$^{+}$ T cells treated with DMSO (blues) or 5 µM AZD6738 (black) (left panel) or proliferating CD8$^{+}$ T cells treated with DMSO (blues) and primary fibroblasts treated with DMSO (reds) (right panel).

(D) DNA combing analyses of CD8⁺ T cells, B16 cells, and fibroblasts treated with ATRi for 1 h. Cells were treated with IdU from 30-40 min and CldU from 40-60 min of the treatment. Mean and SD bars shown. **:P<0.01,****:P<0.0001 by two-tailed, unpaired t-test.

A

| Concentration (ng/mL) | 1,000.00 | 500.00 | 250.00 | 125.00 | 62.50 | 31.25 | 15.62 | 7.81 | 3.90 | 1.95 | Blank | | |
|---|---|---|---|---|---|---|---|---|---|---|---|---|---|
| Dilution | 1:1,000 | 1:2,000 | 1:4,000 | 1:8,000 | 1:16,000 | 1:32,000 | 1:64,000 | 1:128,000 | 1:256,000 | 1:512,000 | Blank | Titer | Coating |
| *107C2-2* | 2.792 | 2.739 | 2.778 | 2.686 | 2.567 | 2.387 | 2.202 | 1.867 | 1.495 | 1.067 | 0.056 | >1:512,000 | A |
| *107C2-2* | 0.054 | 0.057 | 0.060 | 0.055 | 0.052 | 0.057 | 0.066 | 0.052 | 0.055 | 0.058 | 0.057 | <1:1,000 | B |

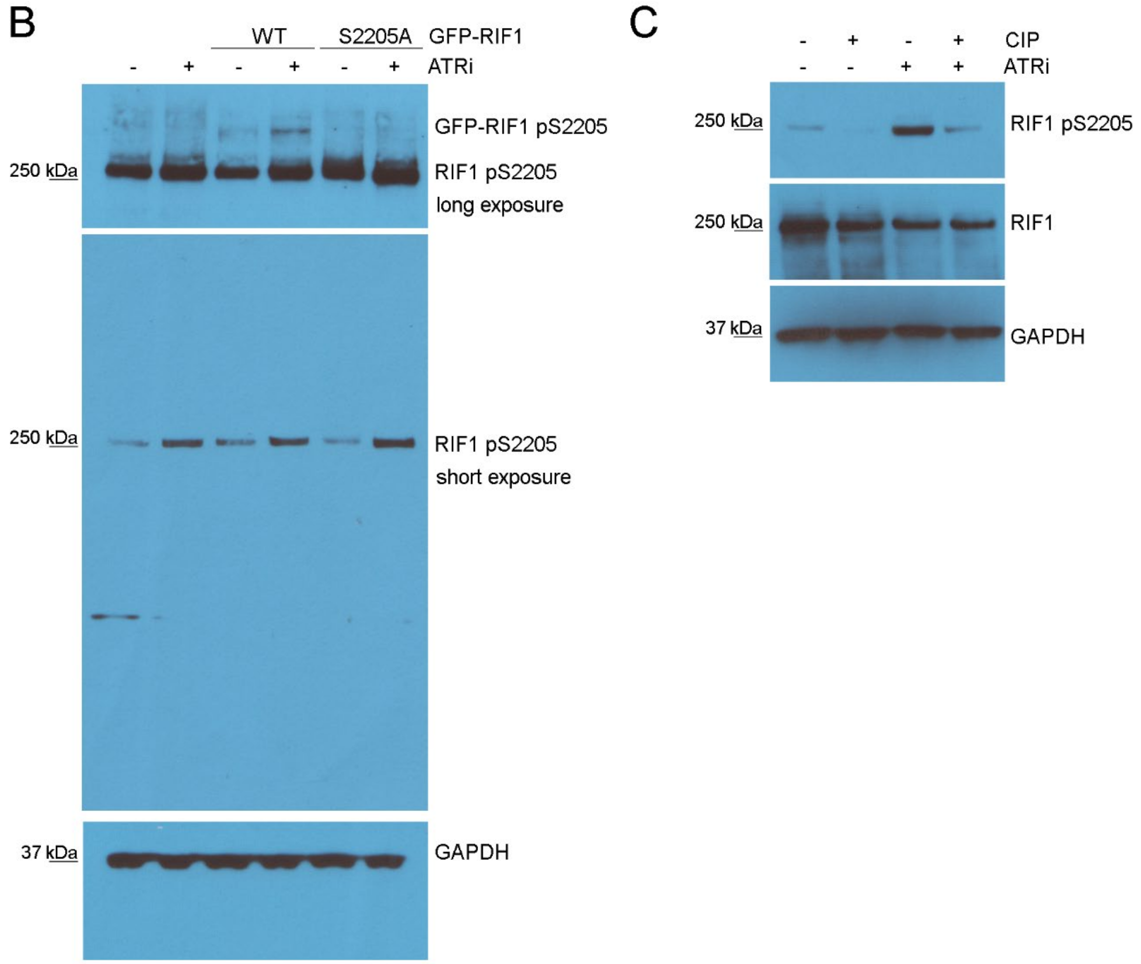


**Figure S5. Rabbit monoclonal antibody was generated against a synthetic peptide CKVRRV(pSer)FADPI, related to Figure 3H.**

(A) ELISA of the hybridoma supernatant shows selectivity against phosphopeptide A: CKVRRV(pSer)FADPI vs. nonphosphopeptide B: CKVRRVSFADPI.

(B) GFP-RIF1 WT (wild-type) or GFP-RIF1 S2205A mutated were expressed in 293T cells. Cells were treated with ATRi for 1 h and whole cell extracts were generated and immunoblotted using the purified rabbit monoclonal antibody from A. Upper panel is a long exposure that shows that GFP-RIF1 WT, but not GFP-RIF1 S2205A mutated, is recognized by the rabbit monoclonal antibody in cells treated with ATRi. Middle panel is a typical exposure that shows that endogenous RIF1 is recognized by the rabbit monoclonal antibody in whole cell extracts of 293T cells treated with ATRi.

(C) Whole cell extracts of 293T cells treated with vehicle or ATRi were treated with calf intestinal phosphatase (CIP). RIF1 is recognized by the rabbit monoclonal antibody in whole cell extracts of 293T cells treated with ATRi and this is reversed by treatment with CIP.

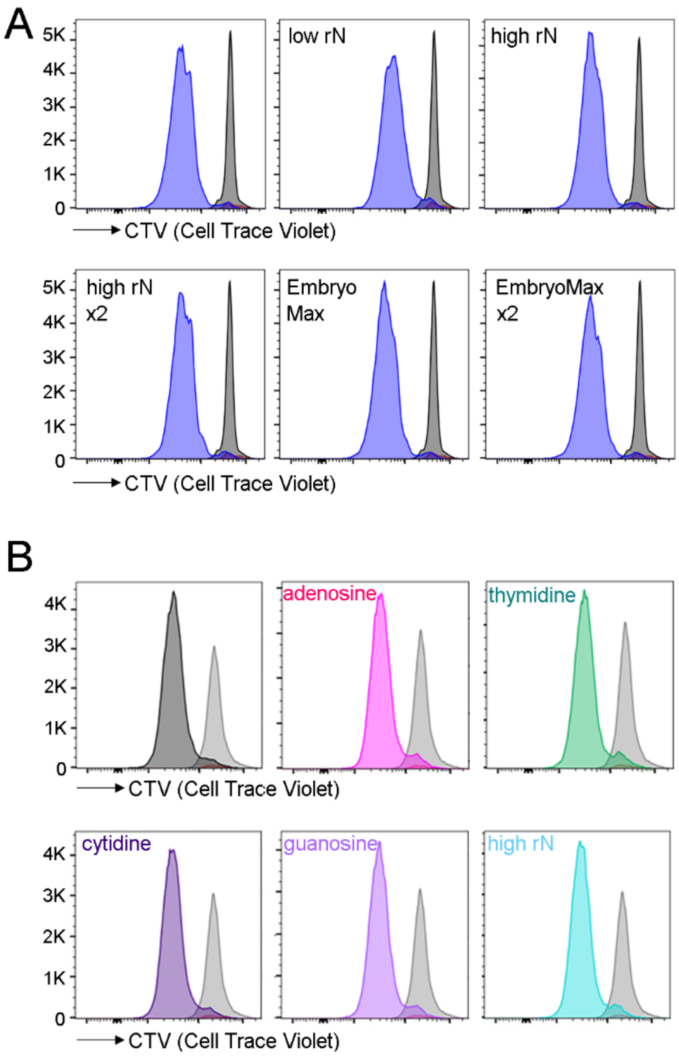


**Figure S6. Cell proliferation in CD8$^+$ T cells treated with nucleosides, related to Figure 4A and Figure 5A.**

(A) Representative overlays of CTV (proliferation) histograms for proliferating CD8$^+$ T cells treated with DMSO and different nucleosides analyzed at 24 h (gray) or 48 h (blue). This is from the experiment shown in Figure 4A. Red histogram represent unactivated samples. CTV histograms are within live, CD44$^{hi}$CD8$^+$ gates.

(B) Representative overlays of CTV (proliferation) histograms for proliferating CD8$^+$ T cells treated with DMSO and different nucleosides analyzed at 24 h (gray) or 48 h (blue). This is from the experiment shown in Figure 5A. Red histogram represent unactivated samples. CTV histograms are within live, CD44$^{hi}$CD8$^+$ gates.

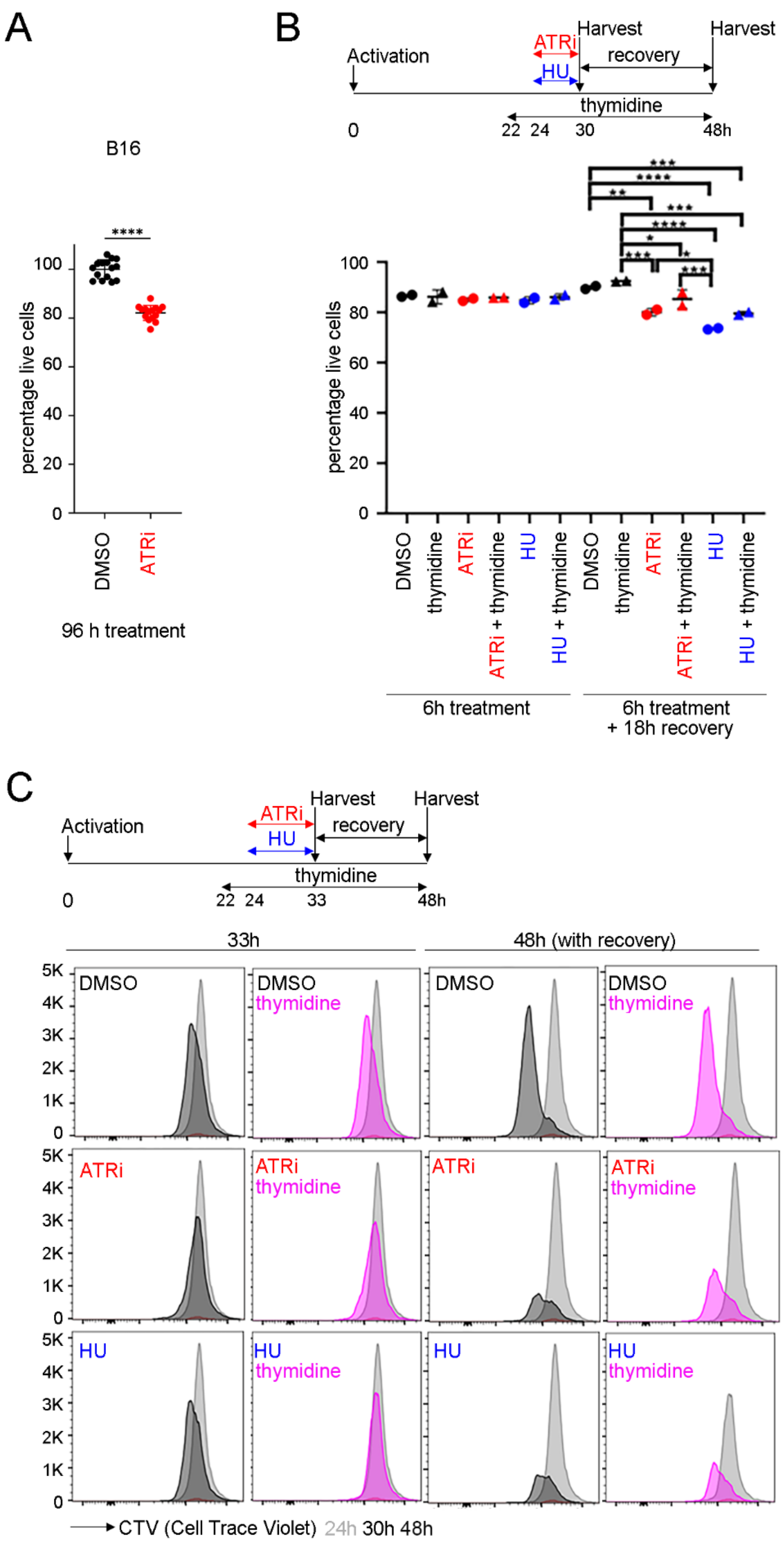
A
B16
percentage live cells
DMSO
ATRi
96 h treatment
B
Activation
ATRi
HU
Harvest
recovery
Harvest
thymidine
0
22 24 30
48h
DMSO
thymidine
ATRi
ATRi + thymidine
HU
HU + thymidine
6h treatment
6h treatment
+ 18h recovery
C
Activation
ATRi
HU
Harvest
recovery
Harvest
thymidine
0
22 24 33 48h
33h
48h (with recovery)
DMSO
thymidine
ATRi
HU
CTV (Cell Trace Violet) 24h 30h 48h

Figure S7

**Figure S7. Survival of B16 cells treated with ATRi and survival and proliferation of CD8$^+$ cells treated with ATRi, HU, and thymidine, related to Figure 5E, Figure 6A, B.**

(A) B16 cells were treated with 5 µM or AZD6738 (ATRi) for 96 h and cell viability was determined using CellTiter-Glo. Data from three independent experiments, each with 5 biological replicates. Mean and SD bars shown. ****:P<0.0001 by two-tailed, unpaired t-test.

(B) CD8$^+$ T cells were treated with 6 µM dT at 22 h and either 5 µM AZD6738 (ATRi) or 5 mM HU at 24 h. At 30 h, cells were re-plated with and without 6 µM dT in the absence of ATRi and HU. At 48 h, the percentage of live CD8$^+$ T cells (eFluor 780$^-$CD8$^+$TCRβ$^+$) was quantitated for the experiment shown in Figure 6A. Mean and SD bars shown. Statistics represent one-way ANOVA with Tukey's multiple comparisons where *:P<0.05,**:P<0.01,***:P<0.001,****:P<0.0001. Brackets not shown for comparisons that were not statistically significant and between DMSO and ATRi treated samples for clarity.

(C) Representative overlays of CTV (proliferation) histograms at 24 h (gray), 33 h or 48 h post stimulation for proliferating CD8$^+$ T cells treated with ATRi or HU and DMSO (black) or thymidine (pink) for the experiment shown in Figure 6B. CTV histograms are within live, CD44$^{hi}$CD8$^+$ gates.

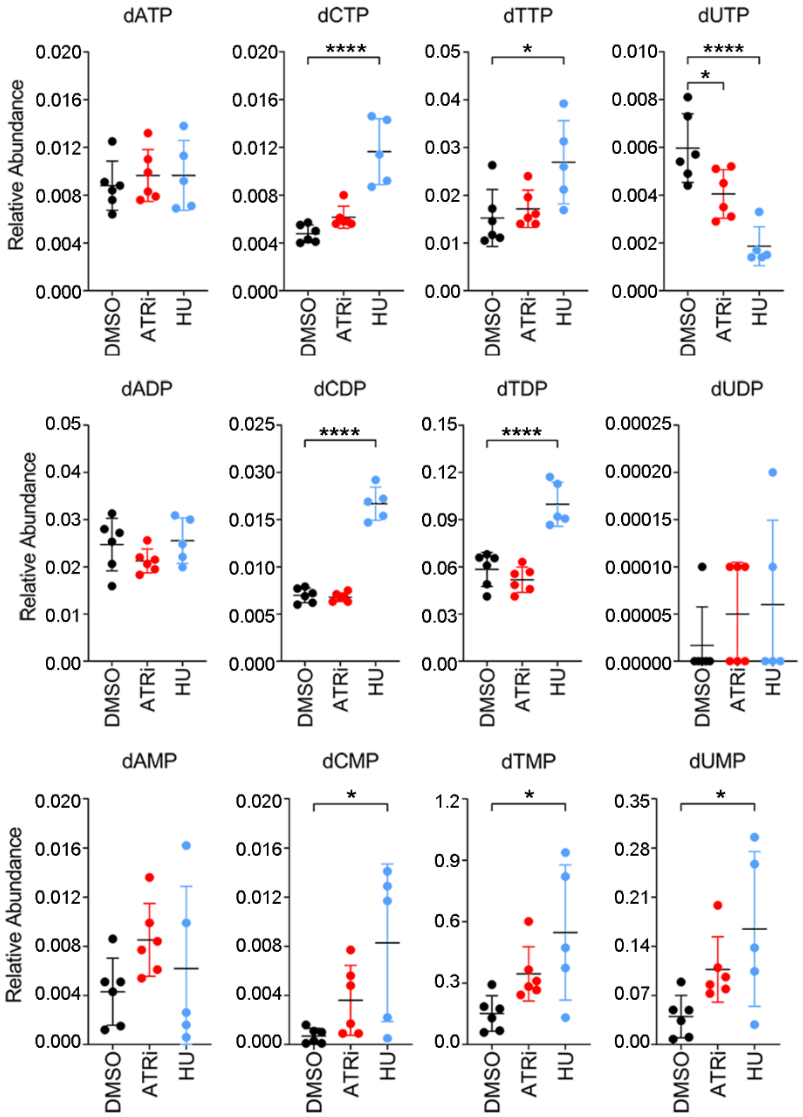


**Figure S8. Quantitation of cellular deoxyribonucleosides in proliferating CD8⁺ T cells treated with DMSO, 5 µM ATRi, or 5 mM HU, related to Figure 6J.**

Mean and SD bars shown. *:P<0.05,****:P<0.0001 by one-way ANOVA with Dunnett's multiple comparisons (DMSO as control).

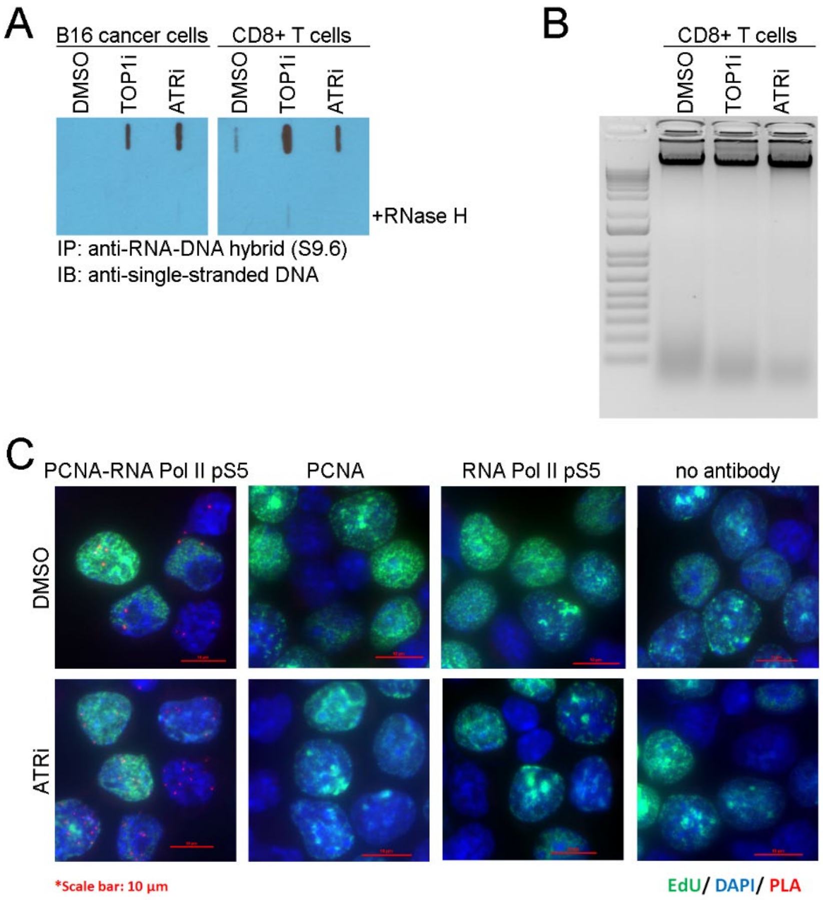


**Figure S9. ATRi-induced R loops and PCN-RNA Pol II pS5 collisions, related to Figure 7C-F.**

(A) B16 and proliferating CD8$^+$ T cells were treated with 10 µM camptothecin (TOP1i) or 5 µM ATRi for 1 h. Genomic DNA was prepared and digested with restriction endonucleases, +/-RNASE H. DNA fragments were immunopurified with anti-RNA-DNA hybrid antibody (S9.6). Immunopurified DNA was denatured and dot blotted using an anti-single stranded DNA antibody.

(B) Genomic DNA prepared from proliferating CD8$^+$ T cells that was digested with restriction endonucleases, +/-RNASE H in Figure S8A. **C.** Proximity ligation assay (PLA) of PCNA and RNA Pol II phosphoserine-5 in proliferating CD8$^+$ T cells.

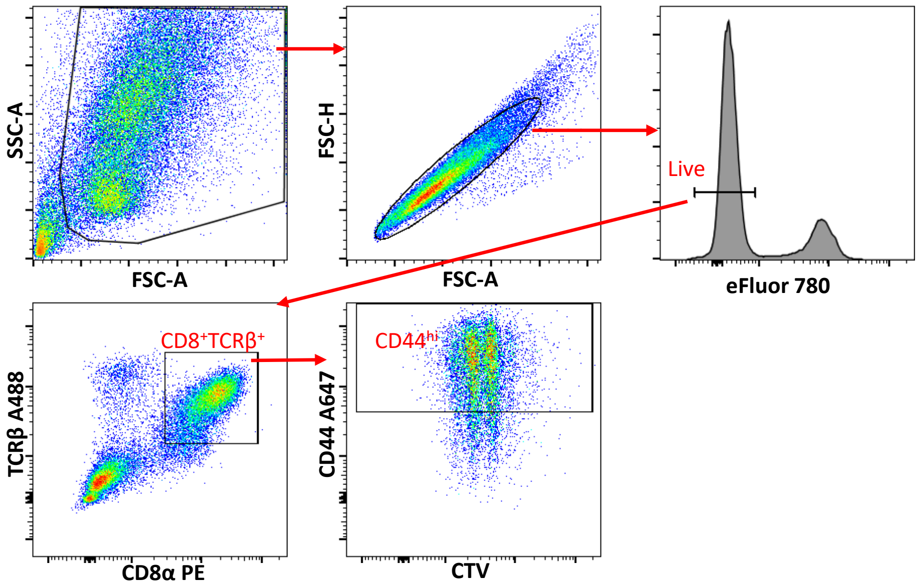


**Figure S10. Flow cytometry gating strategy for CD8$^+$ T cells *ex vivo*, related to Figure 2C-E, Figure 4A, Figure 5A, and Figure 6B.**

Any unstable portions of the run were gated out prior to analysis. *Ex vivo* cultured CD8$^+$ T cells were gated first with scatter gating (FSC-A vs. SSC-A) to exclude debris followed by single cell gating (FSC-A vs. FSC-H) to remove doublets/clumps. For proliferation assay, CD8$^+$ T cells were identified as CD8$^+$TCRβ$^+$ cells within live population. CTV proliferation dye dilution was further characterized in CD44$^{hi}$ population within these cells.

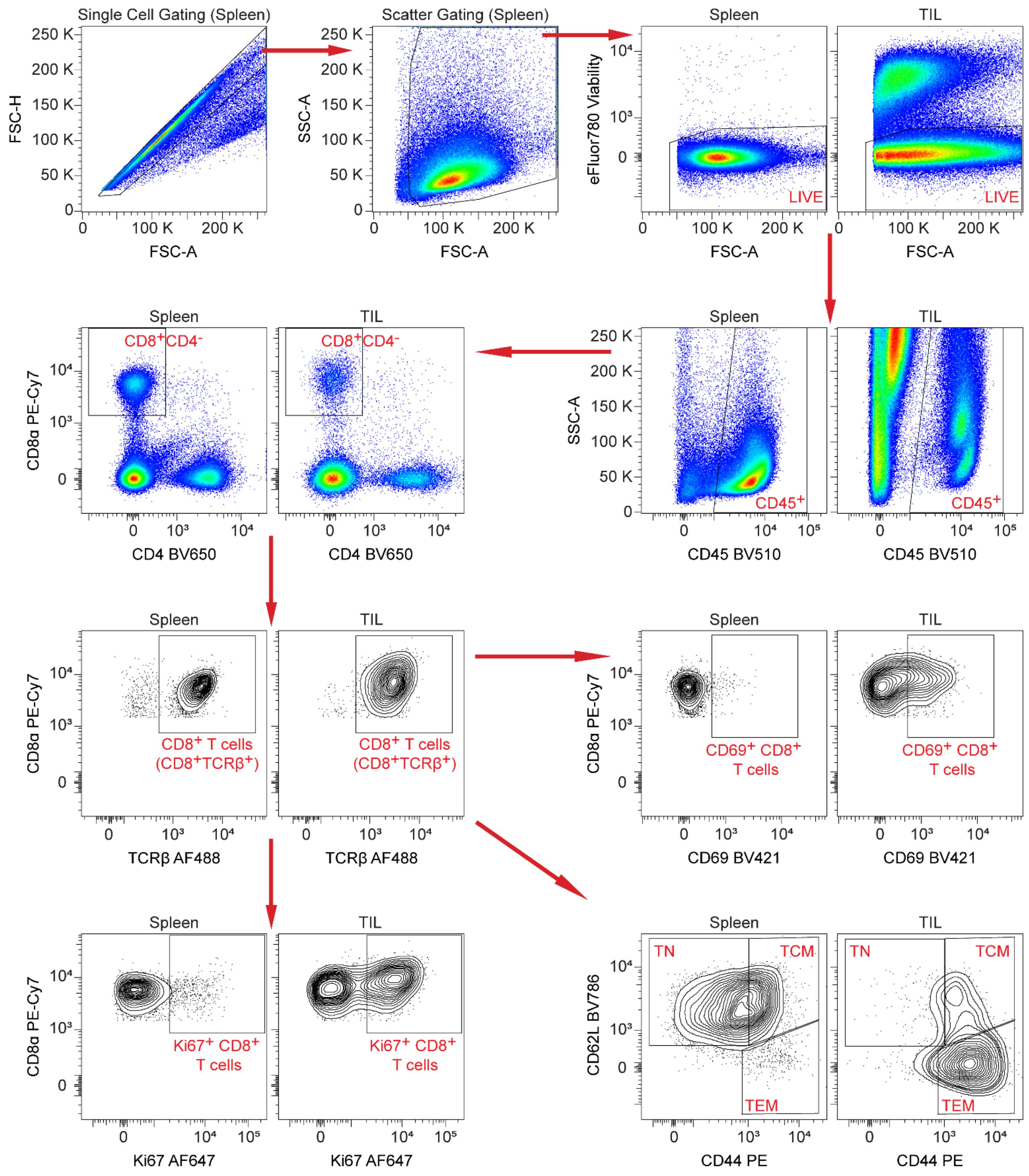

Single Cell Gating (Spleen)
FSC-H
FSC-A
Scatter Gating (Spleen)
SSC-A
Spleen
TIL
eFluor780 Viability
LIVE
CD8α PE-Cy7
CD4 BV650
CD8+CD4-
CD45 BV510
CD45+
CD8+ T cells
(CD8+TCRβ+)
TCRβ AF488
CD69 BV421
CD69+ CD8+
T cells
Ki67 AF647
Ki67+ CD8+
T cells
CD62L BV786
CD44 PE
TN
TCM
TEM


**Figure S11**

**Figure S11. Flow cytometry gating strategy for CD8$^+$ T cell profiling in spleen, TIL, and DLN, related to Figure 2.**

Any unstable portions of the run were gated out prior to analysis. After single cell gating (FSC-A vs. FSC-H) to remove doublets/clumps and scatter gating (FSC-A vs. SSC-A) to exclude debris, CD8$^+$ T cells were identified as CD8$^+$CD4$^-$TCRβ$^+$ cells within the live, CD45$^+$ immune cell population. CD8$^+$ T cells were further profiled to identify proliferating (Ki67$^+$), newly activated (CD69$^+$), naïve (TN, CD62L$^{lo}$CD44$^{lo}$), central memory (TCM, CD62L$^{hi}$CD44$^{hi}$), and effector/effector memory (CD62L$^{lo}$CD44$^{hi}$) CD8$^+$ T cells. Example plots are shown for spleen and TIL for all gating following single cell and scatter gating.
